# Selective Hydroboration–Oxidation of Terminal Alkenes under Flow Conditions

**DOI:** 10.1002/chem.202001650

**Published:** 2020-08-06

**Authors:** Mohamed Elsherbini, Florence Huynh, Alice Dunbabin, Rudolf K. Allemann, Thomas Wirth

**Affiliations:** ^1^ School of Chemistry Cardiff University Main Building, Park Place Cardiff CF10 3AT UK

**Keywords:** alcohols, alkenes, flow chemistry, hydroboration, oxidation

## Abstract

An efficient flow process for the selective hydroboration and oxidation of different alkenes using 9‐borabicyclo(3.3.1)nonane (9‐BBN) allows facile conversion in high productivity (1.4 g h^−1^) of amorpha‐4,11‐diene to the corresponding alcohol, which is an advanced intermediate in the synthesis of the antimalarial drug artemisinin. The in situ reaction of borane and 1,5‐cyclooctadiene using a simple flow generator proved to be a cost efficient solution for the generation of 9‐BBN.

## Graphical abstract

**An efficient and scalable flow protocol** for the selective hydroboration–oxidation of terminal alkenes enabled easy access to dihydroartemisinic alcohol, an advanced intermediate in the synthesis of the antimalarial drug artemisinin.

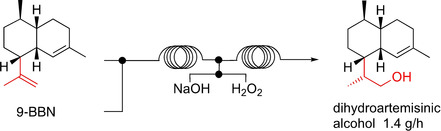

Alkenes are very important entities in organic synthesis due to the diversity and wide availability of alkene substrates and the large spectrum of chemical reactivities of double bonds.^1^ Oxidation of alkenes to the corresponding alcohols is one of the fundamental chemical transformations and the hydroboration–oxidation sequence is an important tool in this context.^2^ In 2015, Souto et al. reported a highly efficient flow method for the hydroboration–oxidation of alkenes.^3^ Their flow protocol presented several clear advantages over batch reactions including milder reaction conditions, better selectivity and a high production rates of up to 120 mmol h^−1^, in addition to the facile continuous processing of the produced biphasic mixture under flow conditions. Despite the efficiency, scalability and simplicity of this protocol, the use of borane poses severe selectivity problems with substrates containing terminal as well as internal double bonds. Limonene, for example, was a challenging substrate that gave diol **2** in only 28 % yield. The problem of a regioselective reaction is alleviated in batch using bulkier hydroborating reagents such as 9‐BBN leading to compound **3** in 77 % yield (Scheme 1).^4^

**Scheme 1:**
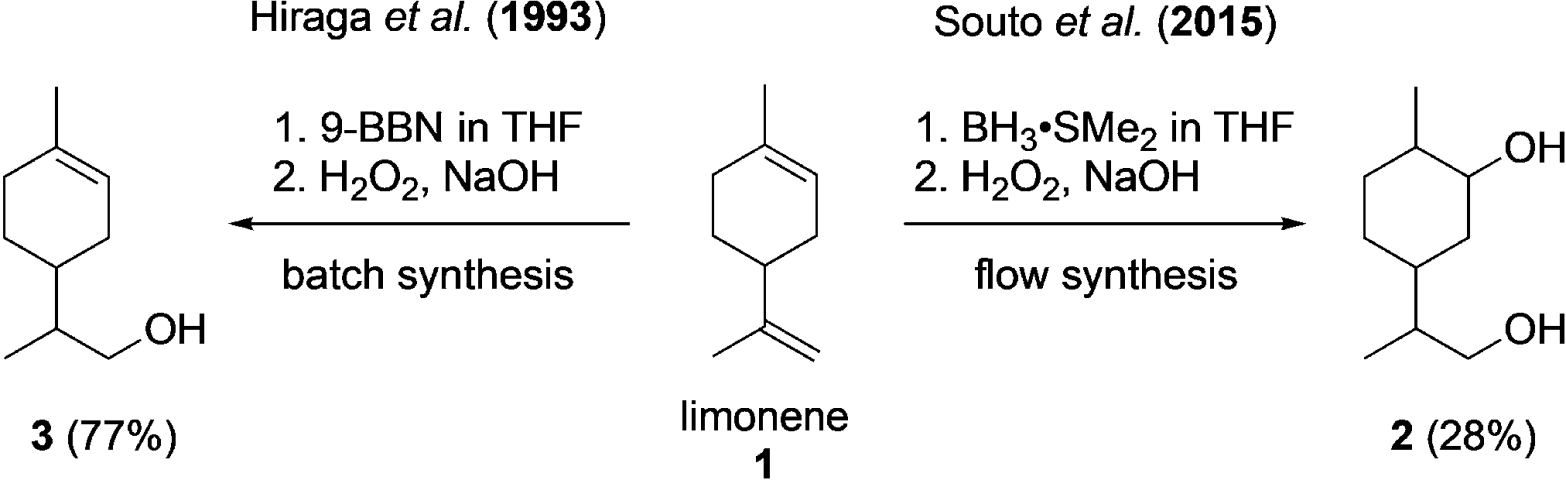
Hydroboration–oxidation of limonene.^3^, ^4^

The selective oxidation of the terminal double bond of amorpha‐4,11‐diene **4** to the corresponding alcohol **5** as shown in Scheme 2 is a key step in the synthesis of the antimalarial drug artemisinin **6**, which can be achieved in batch using 9‐borabicyclo(3.3.1)nonane (9‐BBN).^5^ As part of our ongoing research on the development of an efficient semi‐synthetic approach to artemisinin,^6^ we report here a scalable flow protocol for the selective hydroboration–oxidation of terminal alkenes.

**Scheme 2:**
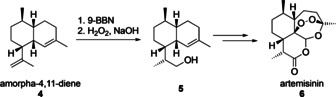
Hydroboration–oxidation of amorpha‐4,11‐diene **4** as a key step in the synthesis of artemisinin **6**.

Initial investigations were based on the optimised reaction conditions for the sequence of hydroboration and oxidation of alkenes in flow as published by Souto et al.^3^ Using a modified reaction setup (Figure 1) and replacing borane by 9‐BBN, the selective oxidation of the terminal alkene of (*R*)‐(+)‐limonene was investigated as a cheap and easily accessible model substrate.

**Figure 1:**
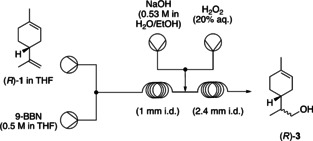
Schematic representation of hydroboration–oxidation flow setup. i.d.=inner diameter.

Under the optimal reaction conditions of Souto^3^ (Table 1, entry 1), the desired alcohol (*R*)‐**3** was formed in only 7 % yield. The hydroboration step takes place in the first reactor (PFA coil, 1 mm i.d.) while the oxidative work‐up proceeds in the second reactor (PFA coil, 2.4 mm i.d.). Coils with a smaller internal diameter for the second reactor resulted in partial blockage of the reactor due to the formation of some solids which was also observed when borane was used as a reagent.^3^ The flow rate of the NaOH solution was adjusted to deliver about 1.7 equivalents to the alkene and the stream of aqueous solution of hydrogen peroxide [20 % (*v*/*v*)] is adjusted to deliver 6.7 equivalents similar to the previously published optimisation.^3^

**Table 1:** Optimisation of hydroboration–oxidation of (*R*)‐**2** in flow.^[a]^

Entry	Limonene (*R*)‐**2** [m]	Flow **A** [mL min^−1^]	Flow **B** [mL min^−1^]	Reactor R1 volume [mL]	Calculated residence time [min]	*T* [°C]	Yield (*R*)‐**3** [%]^[b]^
1	1.0	1.0	2.0 (1 equiv)	2	0.67	r.t.	7
2	0.5	1.0	1.0 (1 equiv)	2	1.0	r.t.	9
3	0.5	1.0	1.0 (1 equiv)	4	2.0	r.t.	15
4	0.25	0.5	0.25 (1 equiv)	4	5.3	r.t.	27
5	0.25	0.5	0.5 (2 equiv)	4	4.0	r.t.	65
6	0.25	0.5	0.5 (2 equiv)	6	6.0	r.t.	69
7	0.25	0.25	0.25 (2 equiv)	4	8.0	r.t.	61
8	0.25	0.5	0.5 (2 equiv)	4	4.0	30	76
9	0.25	0.5	0.5 (2 equiv)	4	4.0	40	92
10	0.25	0.5	0.5 (2 equiv)	4	4.0	50	90
11	0.25	0.5	0.5 (2 equiv)	4	4.0	40	90^[c]^

[a] Reaction conditions: Solvent: THF, flow rate of NaOH (0.53 m) is set to 1.7 equiv, flow rate of H_2_O_2_ (20 % aq.) is set to 6.7 equiv. [b] Determined by ^1^H NMR using 1,3,5‐trimethoxybenzene as the internal standard. [b] Isolated yield, 2.5 mmol scale.

Lowering the concentration of limonene **1** to 0.5 m and increasing the residence time in the first reactor to 1.0 min (entry 2) was not significant. Increasing the reactor volume to 4 mL and hence doubling the residence time to 2 min led to a slight increase in yield (15 %, entry 3). Another increment of the reaction yield (27 %) was obtained by lowering the concentration of limonene to 0.25 m and increasing the residence time to 5.3 min (entry 4). Using two equivalents of 9‐BBN rather than one equivalent (entry 5) led to a dramatic increase of the yield (65 %). Increasing the residence time further by increasing the reactor volume from 4 mL to 6 mL (entry 6) or by lowering the flow rates (entry 7) did not result in an improvement of the reaction outcome. A small improvement (76 %) was obtained by raising the temperature to 30 °C. Another increase of the reaction yield to 92 % was obtained using a reaction temperature of 40 °C (entry 9). Raising the temperature further did not improve the reaction yield (entry 10). All yields were determined by ^1^H NMR using 1,3,5‐trimethoxybenzene as internal standard. Applying the optimum reaction conditions of entry 9 to a reaction on larger scale (2.5 mmol, entry 11) led to the isolation of alcohol **3** in 90 % yield. The reaction was also scaled up further (15 mmol) to prove the efficiency and reliability of the developed flow protocol where 2.1 g (90 %) of alcohol (*R*)‐**3** was obtained in 2 h.

As mentioned above, the main motivation of this work was the development of an efficient flow protocol for the conversion of amorpha‐4,11‐diene **4** to the corresponding alcohol **5**, an advanced intermediate in the synthesis of the antimalarial drug artemisinin **6**. When the optimal conditions (Table 1, entry 9) were used for amorpha‐4,11‐diene **4** as a starting material, alcohol **5** was isolated in 85 % yield. Performing the reaction on 15 mmol scale led to the production of **5** with a production rate of 1.4 g h^−1^ without a reduction in yield. In addition, *epi*‐amorpha‐4,11‐diene^5^ was also successfully converted to the corresponding alcohol **7** in 85 % yield under the same conditions. The method was also applied to other substrates to prove its general applicability (Scheme 3). Both (+)‐valencene and (−)‐β‐pinene gave the corresponding alcohols **8** and **9** in excellent yields of 90 % and 95 %, respectively. Under the same reaction conditions, both terminal double bonds of deca‐1,9‐diene reacted to give diol **10** in 91 % yield. Slightly lower yields were obtained in the case of 5‐bromopent‐1‐ene and styrene where the corresponding alcohols **11** and **12** were obtained in 81 % and 77 % yield, respectively.

**Scheme 3:**
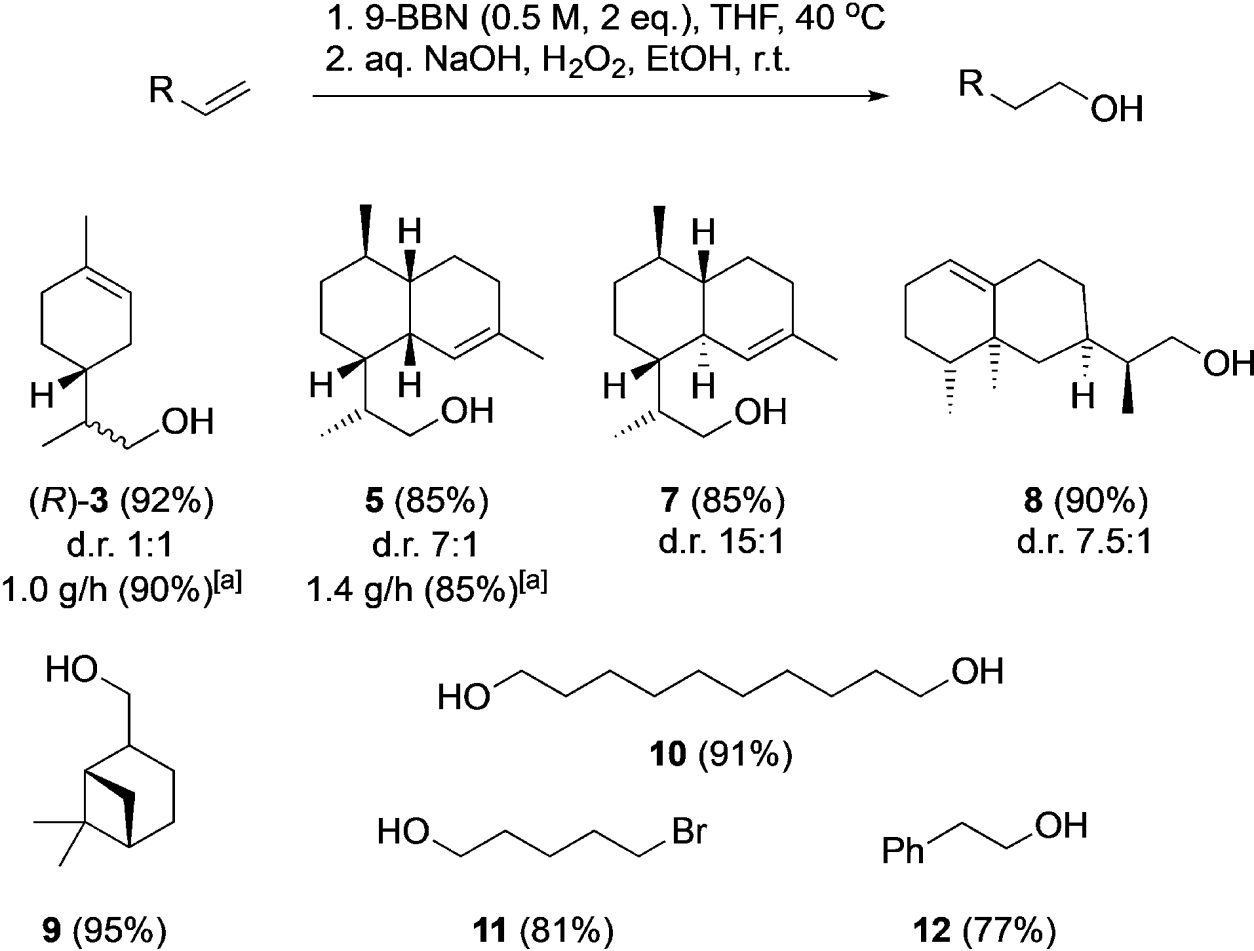
Hydroboration–oxidation of various alkenes under flow conditions. [a] 15 mmol scale.

As a further proof of concept, the 9‐BBN solution was replaced with a flow generator of 9‐BBN from borane (BH_3_⋅THF) and 1,5‐cycloctadiene^7^ (Figure 2) leading to the isolation of alcohol (*R*)‐**3** in 56 % yield. Although the yield of (*R*)‐**3** was lower compared to using commercially available 9‐BBN (Figure 1), the experiment shows clearly the feasibility of the flow generation of 9‐BBN and the potential of a future development of an efficient 9‐BBN generator from cheaper reagents.

**Figure 2:**
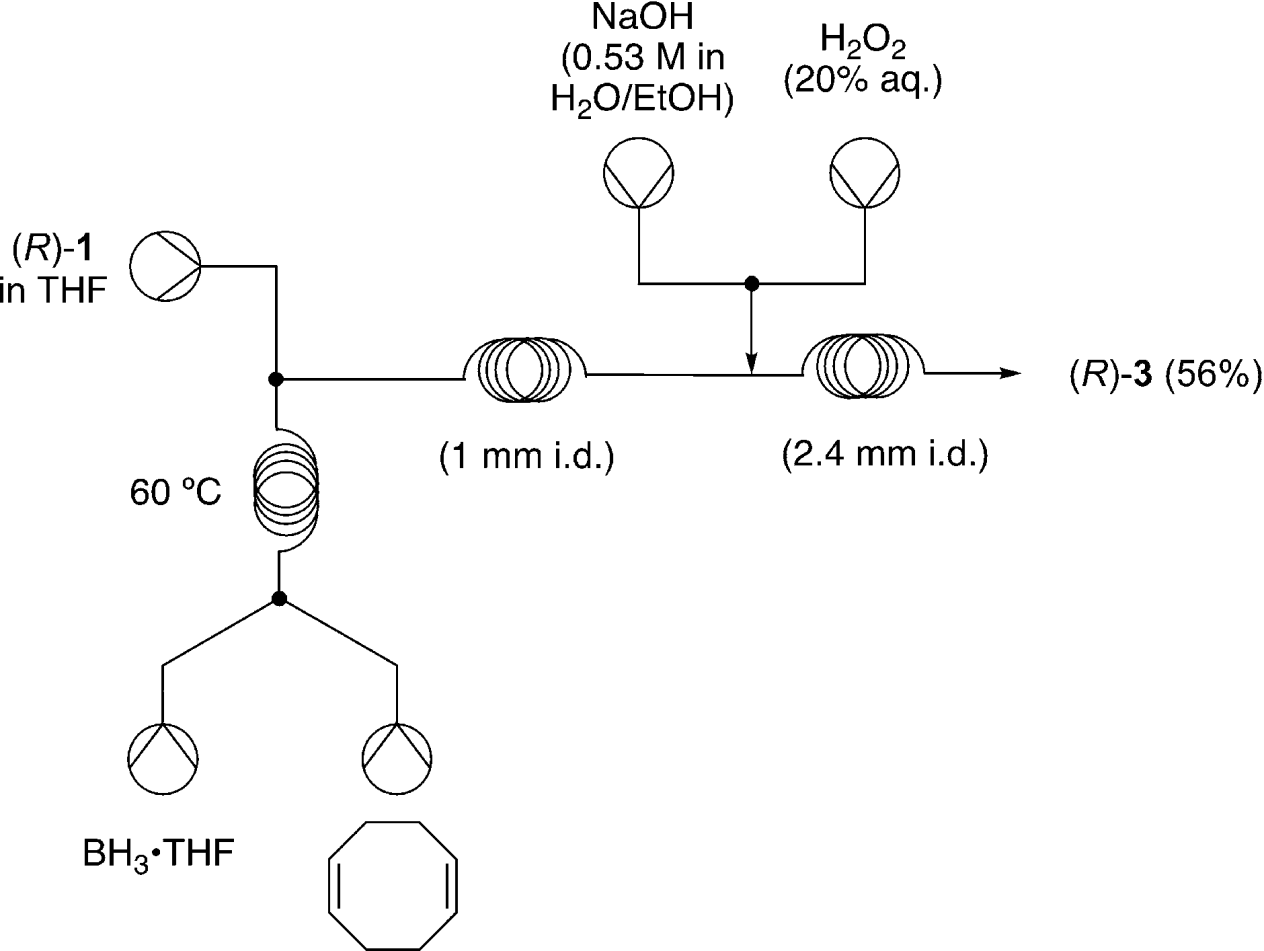
Schematic representation of the flow generation of 9‐BBN coupled to the hydroboration–oxidation sequence of (*R*)‐**3**.

In conclusion, an efficient and scalable protocol for the selective hydroboration–oxidation of terminal alkenes using 9‐BBN under flow conditions was developed. This protocol is particularly useful for substrates containing terminal and internal double bonds such as limonene, amorpha‐4,11‐diene and valencene, where selective functionalisation of the terminal alkene is observed without affecting the internal alkene, a problem that is encountered when borane is used. The method was efficiently applied to the conversion of amorpha‐4,11‐diene **4** to dihydroartemisinic alcohol **5** (1.4 g h^−1^), an important advanced intermediate in the synthesis of the antimalarial drug artemisinin **6**. In addition, the flow generation of 9‐BBN from borane and 1,5‐cyclooctadiene using a simple generator was probed and the preliminary results are promising. Development of an efficient fully integrated generator of 9‐BBN is ongoing in our laboratories.

## Experimental Section

**Synthetic protocol**: Using the reaction setup shown in Figure 1, a solution of alkene (0.25 m) in THF and 9‐BBN (0.5 m) in THF pumped at 0.5 mL min^−1^ each were combined using a T‐piece and reacted in a 4 mL PFA coil (R1, 1 mm i.d.) at 40 °C. A second stream formed by combining a solution of NaOH (0.53 m) in a mixture of water and ethanol (52.5:47.5) at 0.4 mL min^−1^ and an aqueous solution of H_2_O_2_ (20 % *v*/*v*) at 0.1 mL min^−1^ was combined with the outlet of reactor R1 through a T‐piece and the combined solutions reacted at room temperature in a second PFA coil (R2, 2.4 mm i.d., 4.4 mL). The reaction mixture was received in a flask containing saturated aqueous ammonium chloride solution to quench the reaction. The two phases were separated, and the aqueous layer was extracted three times with Et_2_O. The combined organic layers were washed with water then brine and dried over anhydrous MgSO_4_, filtered and evaporated under reduced pressure to give the crude reaction mixture which was then purified by flash column chromatography.

## Conflict of interest

The authors declare no conflict of interest.

## Supporting information

As a service to our authors and readers, this journal provides supporting information supplied by the authors. Such materials are peer reviewed and may be re‐organized for online delivery, but are not copy‐edited or typeset. Technical support issues arising from supporting information (other than missing files) should be addressed to the authors.

Supplementary
