# Supplementary material for: Selective Hydroboration–Oxidation of Terminal Alkenes under Flow Conditions
Source: Chemistry. 2020 Aug 6;26(50):11423–5. doi: 10.1002/chem.202001650 (PMC7540268; doi:10.1002/chem.202001650)

Chemistry–A European Journal

Supporting Information

Selective Hydroboration–Oxidation of Terminal Alkenes under Flow Conditions

Mohamed Elsherbini, Florence Huynh, Alice Dunbabin, Rudolf K. Allemann,* and Thomas Wirth*[a]

General information

All solvents and reagents were used as received without purification or drying. Thin-layer chromatography (TLC) was performed on pre-coated aluminium sheets of Merck silica gel 60 F254 (0.20 mm) and visualized by UV radiation (254 nm) or staining. Automated column chromatography was performed on a Biotage® Isolera Four using Biotage® cartridges SNAP Ultra. ^{1}H NMR and ^{13}C NMR spectra were measured on Bruker DPX 300, 400 or 500 apparatus and were referenced to the residual proton solvent peak (^{1}H: $CDCl_3$, δ 7.26 ppm; DMSO-d_6, δ 2.50 ppm; MeOH-d_4, δ 3.31 ppm) and solvent ^{13}C signal ($CDCl_3$, δ 77.2 ppm, DMSO-d_6, δ 39.5, MeOH-d_4, δ 49.0). Chemical shifts δ were reported in ppm, multiplicity of the signals was declared as followed: s = singlet, d = doublet, t = triplet, q = quartet, quin = quintet, sex = sextet, hep = septet, dd = doublet of doublets, m = multiplet, b = broad; and coupling constants (*J*) in Hertz. Melting points were measured using a Gallenkamp variable heater with samples in open capillary tubes.

Reaction setup A, using commercially available 9-BBN:

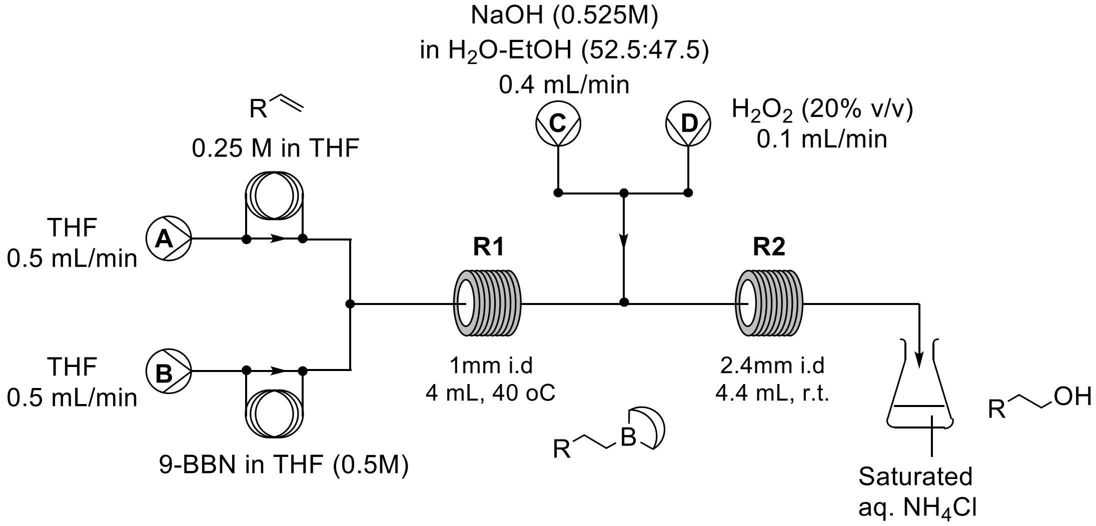

Figure S1. Flow setup **A** for hydroboration/oxidation of alkenes using commercially available 9-BBN.

General method A:

Using the reaction setup shown in in Figure S1, a solution of olefinic substrate (10 mL, 0.25 M, 2.5 mmol) in THF and 9-BBN (10 mL, 0.5 M, 5 mmol) in THF pumped at 0.5 mL/min each were combined using a T-piece and reacted in a 4 mL PFA coil (R1, 1 mm i.d., residence time 4 min) at 40 °C. A second stream formed by combining a solution of NaOH (0.525 M) in a mixture of water and ethanol (52.5:47.5) running at 0.4 mL/min and aqueous solution of H_2O_2 (20% v/v, 0.1 mL/min) was combined with the outlet of reactor R1 via a T-piece and the combined streams reacted at room temperature in a second PFA coil (R2, 2.4 mm i.d., 4.4 mL, residence time 2.93 min). The reaction mixture was received in a flask containing saturated aqueous ammonium chloride solution to quench the reaction. The two phases were separated, and the aqueous layer was extracted with Et_2O (3 x 25 mL). The combined organic layers were washed with water then brine and dried over anhydrous

$MgSO_4$, filtered and evaporated under reduced pressure to give the crude reaction mixture which was then purified by automated column chromatography on a Biotage® Isolera Four, using Biotage® cartridges SNAP Ultra 25 g, applying eluent gradient 5-40% EtOAc/hexane.

Larger scale experiments:

Larger scale reactions were performed using the same reaction setup (Figure S1) and following general method A, but using 60 mL of substrate solution (15 mmol).

Reaction setup B, using in situ generated 9-BBN:

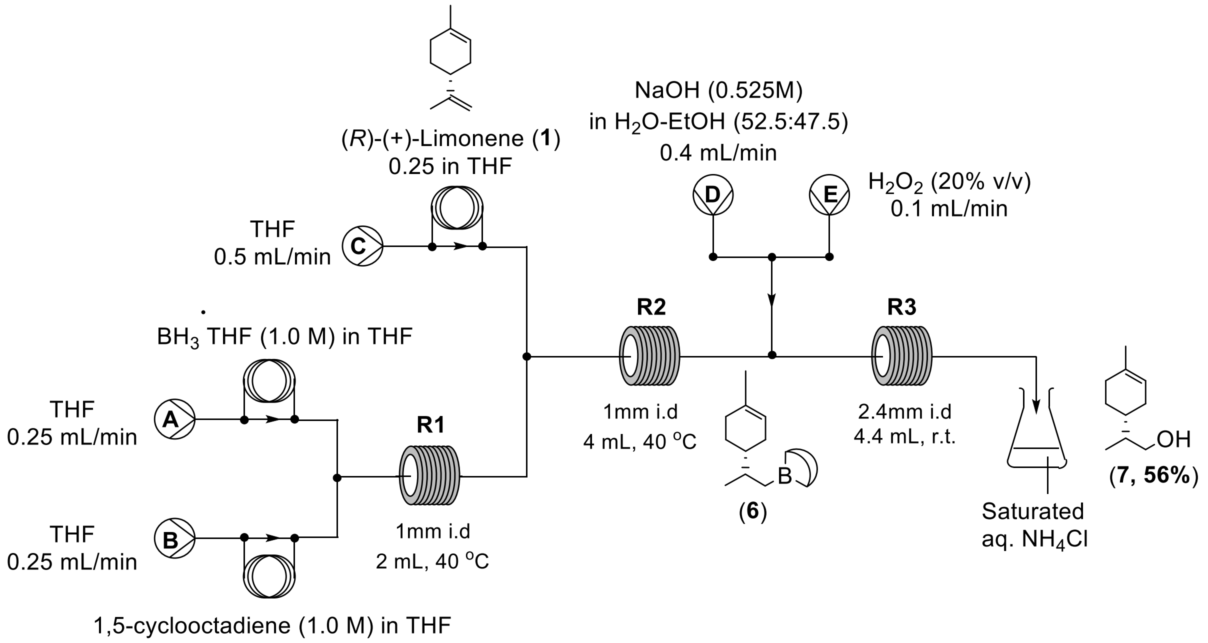

Figure S2. Flow setup **B** for hydroboration/oxidation of (*R*)-(+)-limonene using in situ generated 9-BBN.

General method B:

Using the reaction setup shown in in Figure S2, a solution of (*R*)-(+)-limonene (10 mL, 0.25 M, 2.5 mmol, 0.5 mL/min) in THF and a solution of 9-BBN generated in situ by the reaction of borane (1.0 M, 0.25 mL/min) in THF and 1,5-cyclooctadiene (1.0 M, 0.25 mL/min) in THF were combined using a T-piece and reacted in a 4 mL PFA coil (R1, 1 mm i.d., residence time 4 min) at 40 ºC. A second stream formed by combining a solution of NaOH (0.525 M) in a mixture of water and ethanol (52.5:47.5) running at 0.4 mL/min and aqueous solution of H_2O_2 (20% v/v, 0.1 mL/min) was combined with the outlet of reactor R2 via a T-piece and the combined streams reacted at room temperature in a second PFA coil (R2, 2.4 mm i.d., 4.4 mL, residence time 2.93 min). The reaction mixture was received in a flask containing saturated aqueous ammonium chloride solution to quench the reaction. The two phases were separated, and the aqueous layer was extracted with Et_2O (3 x 25 mL). The combined organic layers were washed with water then brine and dried over anhydrous $MgSO_4$, filtered and evaporated under reduced pressure to give the crude reaction mixture which was then purified by automated column chromatography on a Biotage® Isolera Four, using Biotage® cartridges SNAP Ultra 25g, applying eluent gradient 5-40% EtOAc/hexane.

(*R*)-(+)-(4-Methylcyclohex-3-en-1-yl)propan-1-ol (3):

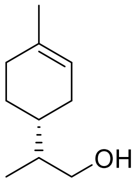

Colourless oil, 347 mg, 2.25 mmol, 90% (1:1 d.r.), $[\alpha]_D^{20} + 76.92$ (c 0.416, $CHCl_3$). ^{1}H NMR (300 MHz, $CDCl_3$): δ = 5.35 (d, J = 1.2 Hz, 1H), 3.63 (ddd, J = 10.6, 5.0, 2.4 Hz, 1H), 3.47 (dd, J = 10.5, 6.5 Hz, 1H), 2.09 – 1.83 (m, 3H), 1.82 – 1.65 (m, 2H), 1.62 (s, 3H), 1.60 – 1.40 (m, 3H), 1.27 (tdd, J = 23.7, 11.3, 5.9 Hz, 1H), 0.89 (d, J = 6.7 Hz, 3H) ppm.^{13}C NMR (75 MHz, $CDCl_3$): δ = 134.2, 134.1, 120.8, 120.7, 66.5, 66.4, 40.2, 40.1, 35.3, 35.2, 30.8, 30.7, 29.9, 27.7, 27.3, 25.5, 23.60, 23.58, 13.8, 13.3 ppm. Spectral data are in good agreement with literature.[1,2]

(*R*)-2-((1*R*,4*R*,4a*S*,8a*S*)-4,7-Dimethyl-1,2,3,4,4a,5,6,8a-octahydronaphthalen-1-yl)propan-1-ol (5):

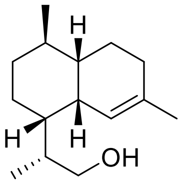

White solid, 473 mg, 2.13 mmol, 85% (7:1 d.r.), m.p.: 64–65 ºC, $[\alpha]_D^{20} - 8.3$ (c 0.96, $CHCl_3$). ^{1}H NMR (500 MHz, $CDCl_3$): δ = 5.22 (dd, J = 11.5, 1.4 Hz, 1H), 3.72 (ddd, J = 20.5, 10.6, 3.3 Hz, 1H), 3.52 (dd, J = 10.6, 6.3 Hz, 1H), 2.57 – 2.34 (m, 1H), 1.98 – 1.85 (m, 2H), 1.85 – 1.75 (m, 1H), 1.70 – 1.49 (m, 7H), 1.47 – 1.35 (m, 1H), 1.28 – 1.14 (m, 3H), 1.04 – 1.00 (m, 1H), 0.99 (d, J = 6.8 Hz, 3H), 0.98 – 0.89 (m, 1H), 0.86 (d, J = 6.5 Hz, 3H) ppm.

Major isomer: ^{13}C NMR (126 MHz, $CDCl_3$): δ = 135.3, 120.8, 67.0, 42.8, 42.2, 37.7, 36.8, 35.8, 27.8, 26.8, 26.5, 26.0, 24.0, 20.0, 15.1 ppm. Spectral data are in good agreement with literature.[3]

Minor isomer: ^{13}C NMR (126 MHz, $CDCl_3$): δ = 135.4, 120.9, 66.2, 43.8, 42.1, 38.2, 36.2, 36.0, 27.9, 26.8, 26.2, 25.9, 24.0, 20.0, 15.9 ppm.

(*R*)-2-((1*R*,4*R*,4a*S*,8a*R*)-4,7-Dimethyl-1,2,3,4,4a,5,6,8a-octahydronaphthalene-1-yl)propan-1-ol (7):

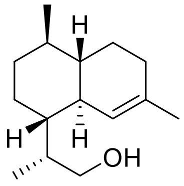

White solid, 473 mg, 2.13 mmol, 85% (15:1 d.r.), m.p.: 61–62 ºC, $[\alpha]_D^{20} - 81.2\ (c\ 1.0, CHCl_3)$. ^{1}H NMR (500 MHz, $CDCl_3$) δ = 5.66 – 5.50 (m, 1H), 3.75 (dt, *J* = 10.1, 4.9 *Hz*, 1H), 3.50 – 3.35 (m, 1H), 2.09 – 1.83 (m, 3H), 1.68 – 1.59 (m, 6H), 1.57 (s, 1H), 1.55 – 1.34 (m, 4H), 1.20 (t, *J* = 5.4 *Hz*, 1H), 1.15 – 1.00 (m, 2H), 0.97 (d, *J* = 7.0 *Hz*, 3H), 0.90 (d, *J* = 6.7 *Hz*, 3H) ppm. ^{13}C NMR (126 MHz, $CDCl_3$) δ = 134.5, 125.4, 65.5, 43.1, 40.1, 39.9, 36.3, 35.7, 31.3, 30.0, 27.9, 23.9, 19.7, 17.1, 15.8 ppm. Spectral data are in good agreement with literature.[4]

(*S*)-2-((2*R*,8*R*,8a*S*)-8,8a-Dimethyl-1,2,3,4,6,7,8,8a-octahydronaphthalen-2-yl)propan-1-ol (8):

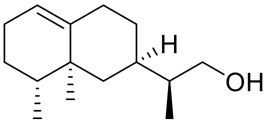

Colourless oil, 500 mg, 2.25 mmol, 90% (7.5:1 d.r.), $[\alpha]_D^{20} + 90.91\ (c\ 0.22, CHCl_3)$. IR ν = 3350 (br), 2956, 2918, 2875, 1654 cm^{-1}. ^{1}H NMR (500 MHz, $CDCl_3$): δ = 5.35 – 5.22 (m, 1H), 3.69 – 3.55 (m, 1H), 3.48 (dd, *J* = 10.5, 6.9 Hz, 1H), 2.32 – 2.16 (m, 1H), 2.06 (ddd, *J* = 13.9, 4.1, 2.5 Hz, 1H), 2.02 – 1.87 (m, 2H), 1.81 – 1.58 (m, 4H), 1.52 – 1.34 (m, 4H), 1.33 – 1.21 (m, 1H), 1.10 – 0.96 (m, 1H), 0.95 – 0.80 (m, 9H) ppm. ^{13}C NMR (126 MHz, $CDCl_3$): δ = 143.7, 143.7, 120.0 (2C), 66.6, 66.4, 44.3, 42.3, 41.2, 40.86, 40.8, 38.0, 37.8, 34.9, 34.5, 33.0, 32.8, 32.2, 29.9, 27.3 (2C), 26.0, 18.7, 15.8 (2C), 13.8, 13.2 ppm.

((1*S*,5*S*)-6,6-Dimethylbicyclo[3.1.1]heptan-2-yl)methanol (9):

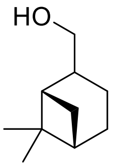

Colourless oil, 386 mg, 2.38 mmol, 95%, $[\alpha]_D^{20} - 26.7\ (c\ 0.15, CHCl_3)$. ^{1}H NMR (400 MHz, $CDCl_3$): δ = 3.64 – 3.50 (m, 2H), 2.44 – 2.31 (m, 1H), 2.29 – 2.18 (m, 1H), 2.06 – 1.99 (m, 1H), 1.98 – 1.84 (m, 4H), 1.52 – 1.40 (m, 1H), 1.35 (br s, 1H), 1.19 (s, 3H), 0.97 (s, 3H), 0.94 (d, *J* = 9.6 Hz, 1H) ppm. ^{13}C NMR (101 MHz, $CDCl_3$): δ = 68.0, 44.7, 43.1, 41.7, 38.8, 33.3, 28.1, 26.2, 23.5, 18.9 ppm. Spectral data are in good agreement with literature.[5]

Decane-1,10-diol (10):

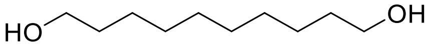

Colourless oil, 397 mg, 2.28 mmol, 91%. ^{1}H NMR (400 MHz, $CDCl_3$) δ = 3.64 (t, *J* = 6.6 Hz, 4H), 1.65 – 1.46 (m, 6H), 1.43 – 1.11 (m, 12H) ppm.^{13}C NMR (101 MHz, $CDCl_3$) δ = 63.3, 33.0, 29.5, 29.4, 25.9 ppm. Spectral data are in good agreement with literature.[6] The product after purification contained *n*-hexane (from the eluent system) and all our trials of the complete removal of the *n*-

hexane were unsuccessful, probably due to the interaction with the long hydeocarbon chain of diol **10**.

5-Bromopentan-1-ol (11):

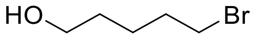

Colourless oil, 418 mg, 2.03 mmol, 81%. ^{1}H NMR (400 MHz, $CDCl_3$): δ = 3.66 (t, *J* = 6.3 Hz, 2H), 3.42 (t, *J* = 6.8 Hz, 2H), 1.90 (dt, *J* = 14.1, 6.9 Hz, 2H), 1.65 – 1.47 (m, 4H), 1.42 (br s, 1H) ppm. ^{13}C NMR (101 MHz, $CDCl_3$): δ = 62.8, 33.8, 32.7, 31.9, 24.6 ppm. Spectral data are in good agreement with literature.[7]

2-Phenylethan-1-ol (12):

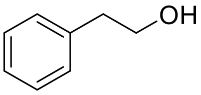

Colourless oil, 236 mg, 1.93 mmol, 77%. ^{1}H NMR (500 MHz, $CDCl_3$): δ = 7.35 – 7.29 (m, 2H), 7.26 – 7.21 (m, 3H), 3.87 (t, *J* = 6.6 Hz, 2H), 2.88 (t, *J* = 6.6 Hz, 2H), 1.44 (s, 1H) ppm. ^{13}C NMR (126 MHz, $CDCl_3$): δ = 138.6, 129.2, 128.7, 126.6, 63.8, 39.3 ppm. Spectral data are in good agreement with literature.[8]

References

[1] S. Serra, C. Fuganti, F. G. Gatti, *Eur. J. Org. Chem.* **2008**, 1031–1037.

[2] Y. Hiraga, H. Danjo, T. Ito, T. Suga, *J. Label. Compd. Radiopharm.* **1993**, *33*, 733–737.

[3] G. Schwertz, A. Zanetti, M. Nascimento de Oliveira, M. A. Gomez Fernandez, F. Dioury, J. Cossy, Z. Amara, *Tetrahedron* **2019**, *75*, 743–748.

[4] J. S. Yadav, B. Thirupathaiah, P. Srihari, *Tetrahedron* **2010**, *66*, 2005–2009.

[5] P. Pommerening, M. Oestreich, *Eur. J. Org. Chem.* **2019**, 7240–7246.

[6] Y. Sawama, M. Masuda, S. Asai, R. Goto, S. Nagata, S. Nishimura, Y. Monguchi, H. Sajiki, *Org. Lett.* **2015**, *17*, 434–437.

[7] K. Antien, A. Lacambra, F. P. Cossío, S. Massip, D. Deffieux, L. Pouységu, P. A. Peixoto, S. Quideau, *Chem. Eur. J.* **2019**, *25*, 11574–11580.

[8] Z. Shao, R. Zhong, R. Ferraccioli, Y. Li, Q. Liu, *Chin. J. Chem.* **2019**, *37*, 1125–1130.

(*R*)-(+)-(4-Methylcyclohex-3-en-1-yl)propan-1-ol (**3**), ^{1}H NMR

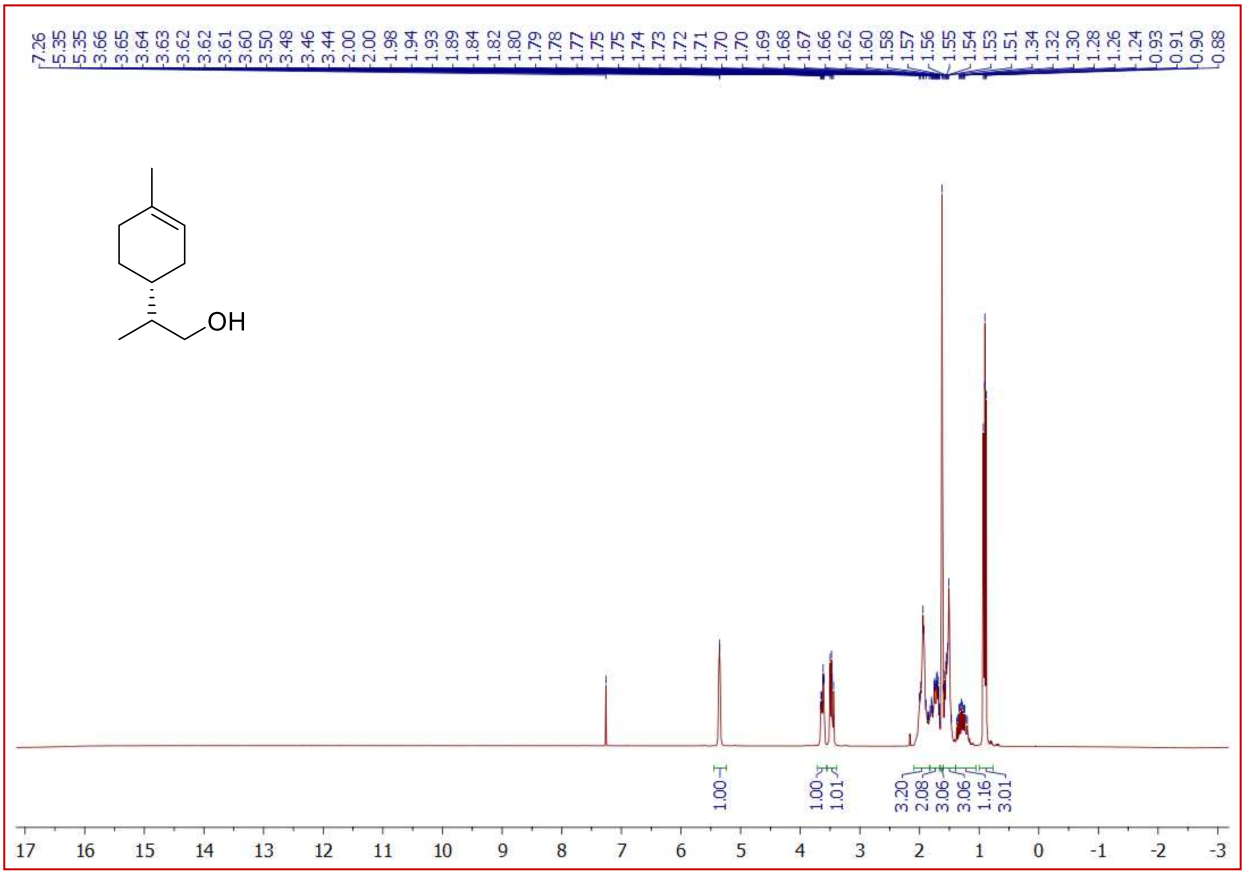

(*R*)-(+)-(4-Methylcyclohex-3-en-1-yl)propan-1-ol (**3**), ^{13}C NMR

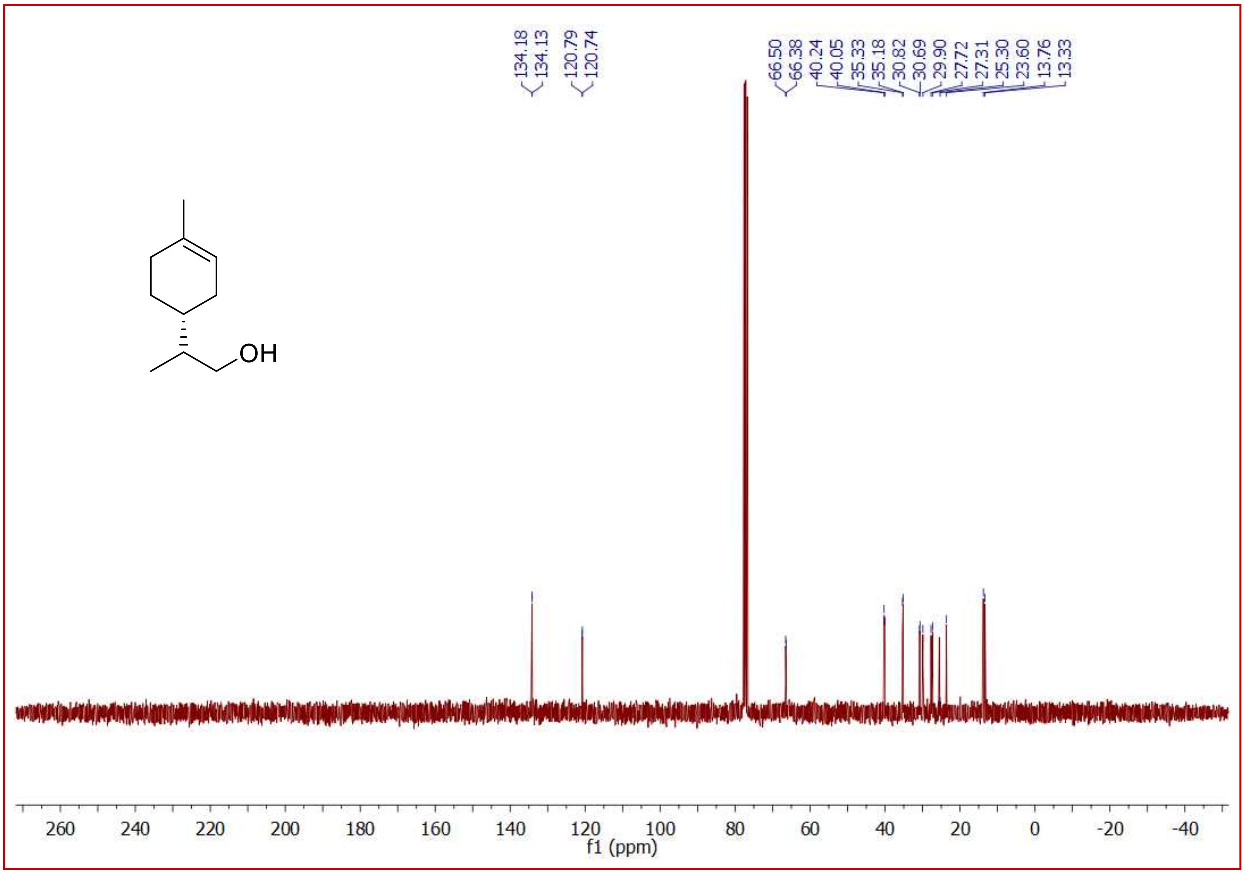

(*R*)-2-((1*R*,4*R*,4a*S*,8a*S*)-4,7-Dimethyl-1,2,3,4,4a,5,6,8a-octahydronaphthalen-1-yl)propan-1-ol (**5**), ^{1}H NMR

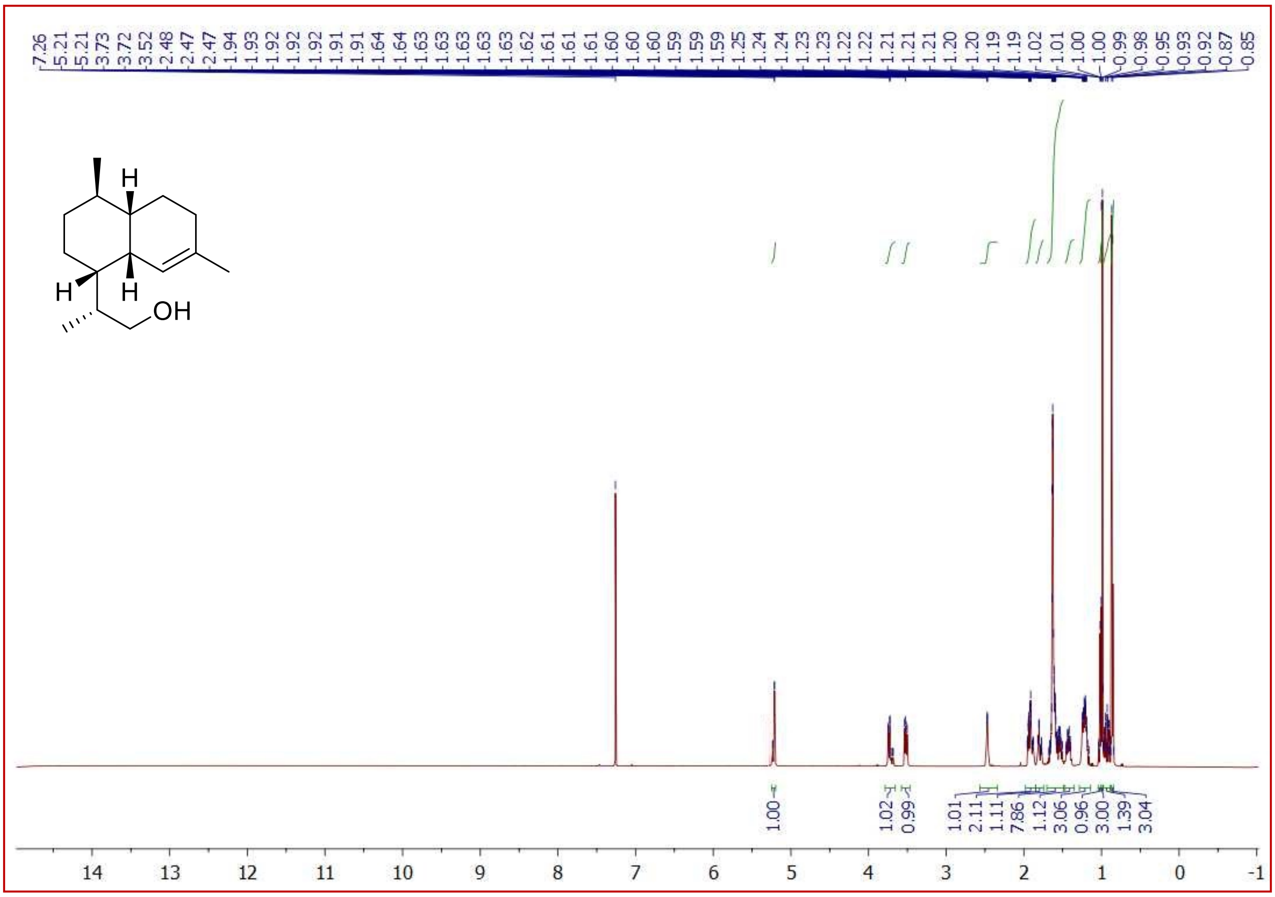

(*R*)-2-((1*R*,4*R*,4a*S*,8a*S*)-4,7-Dimethyl-1,2,3,4,4a,5,6,8a-octahydronaphthalen-1-yl)propan-1-ol (**5**), ^{13}C NMR

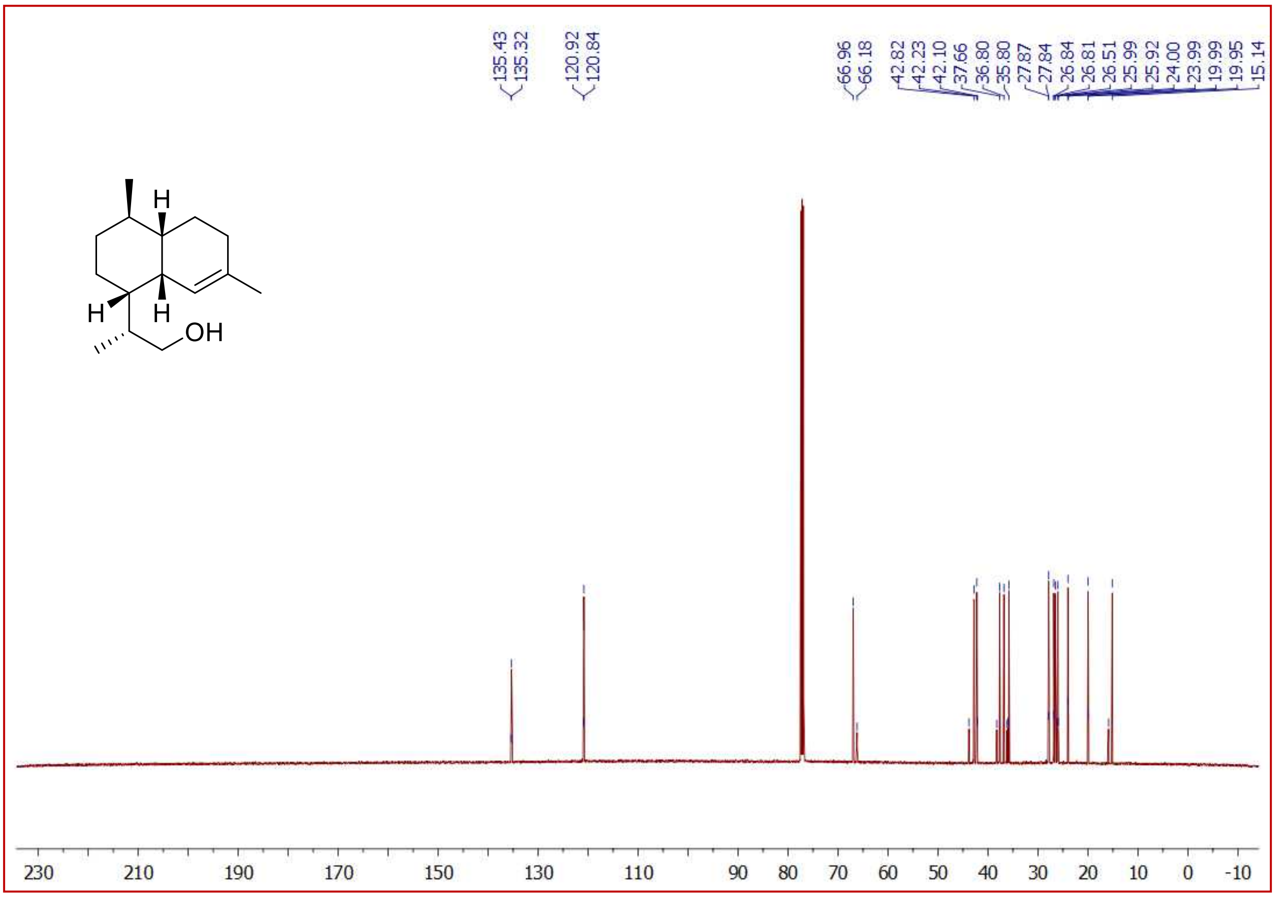

(*R*)-2-((1*R*,4*R*,4a*S*,8a*R*)-4,7-Dimethyl-1,2,3,4,4a,5,6,8a-octahydronaphthalene-1-yl)propan-1-ol (**7**), ^{1}H NMR

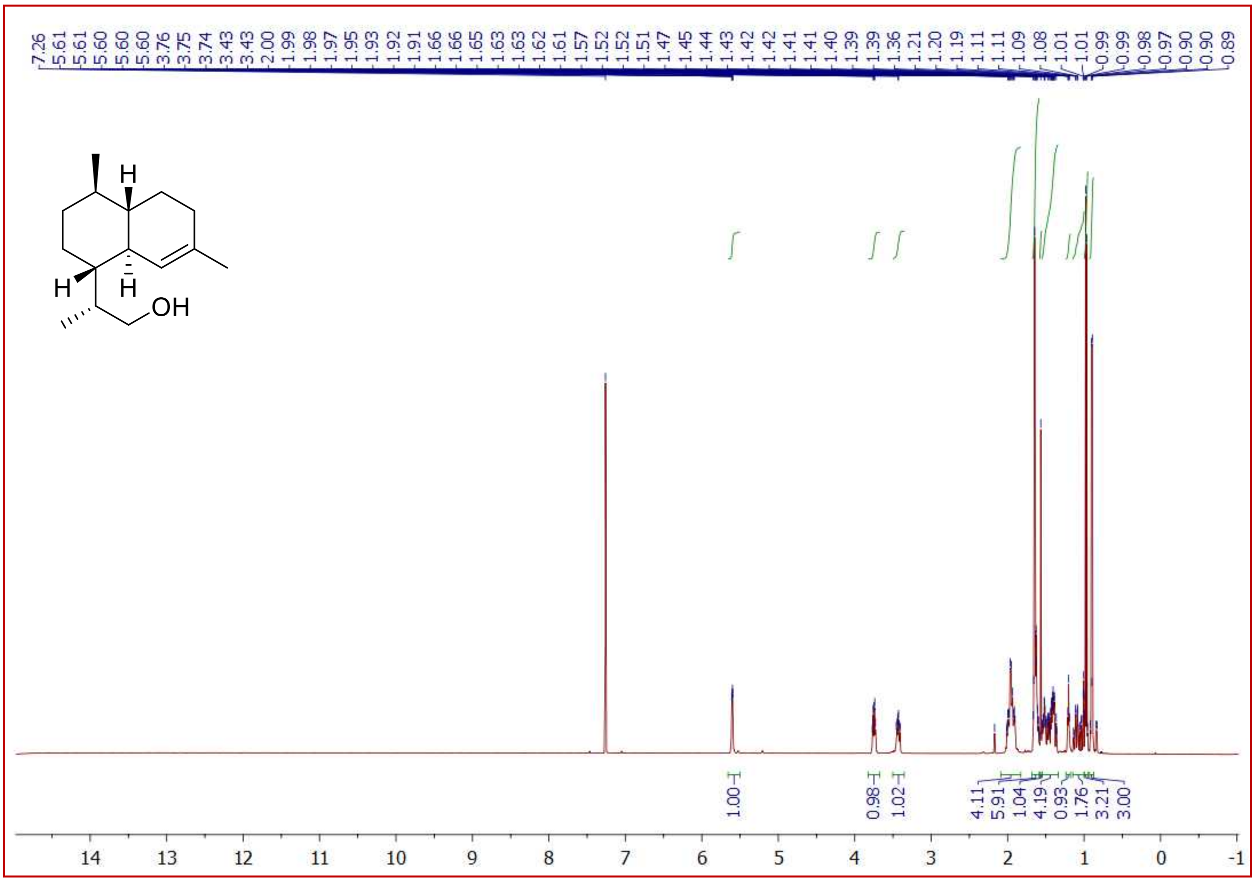

(*R*)-2-((1*R*,4*R*,4a*S*,8a*R*)-4,7-Dimethyl-1,2,3,4,4a,5,6,8a-octahydronaphthalene-1-yl)propan-1-ol (**7**), ^{13}C NMR

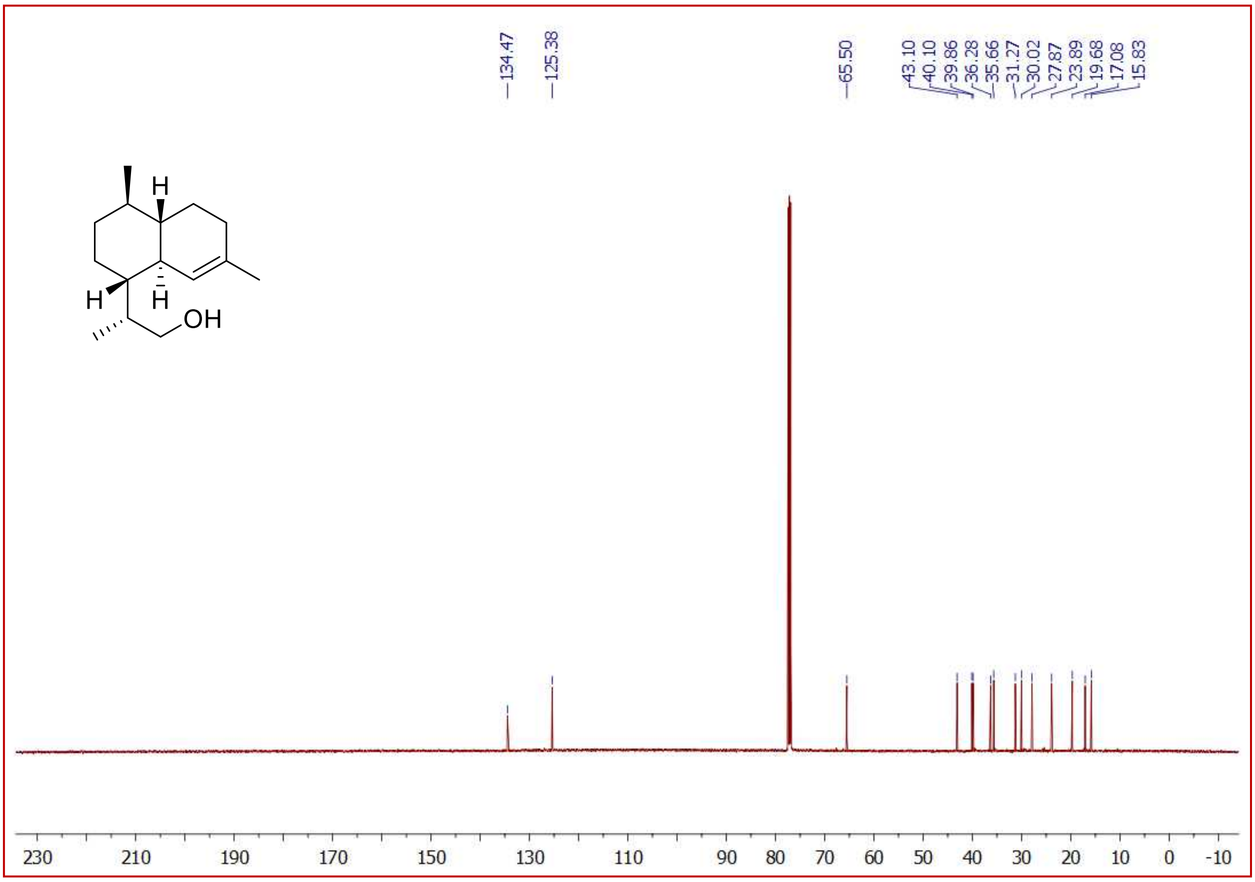

(*S*)-2-((2*R*,8*R*,8a*S*)-8,8a-Dimethyl-1,2,3,4,6,7,8,8a-octahydronaphthalen-2-yl)propan-1-ol (**8**), ^{1}H NMR

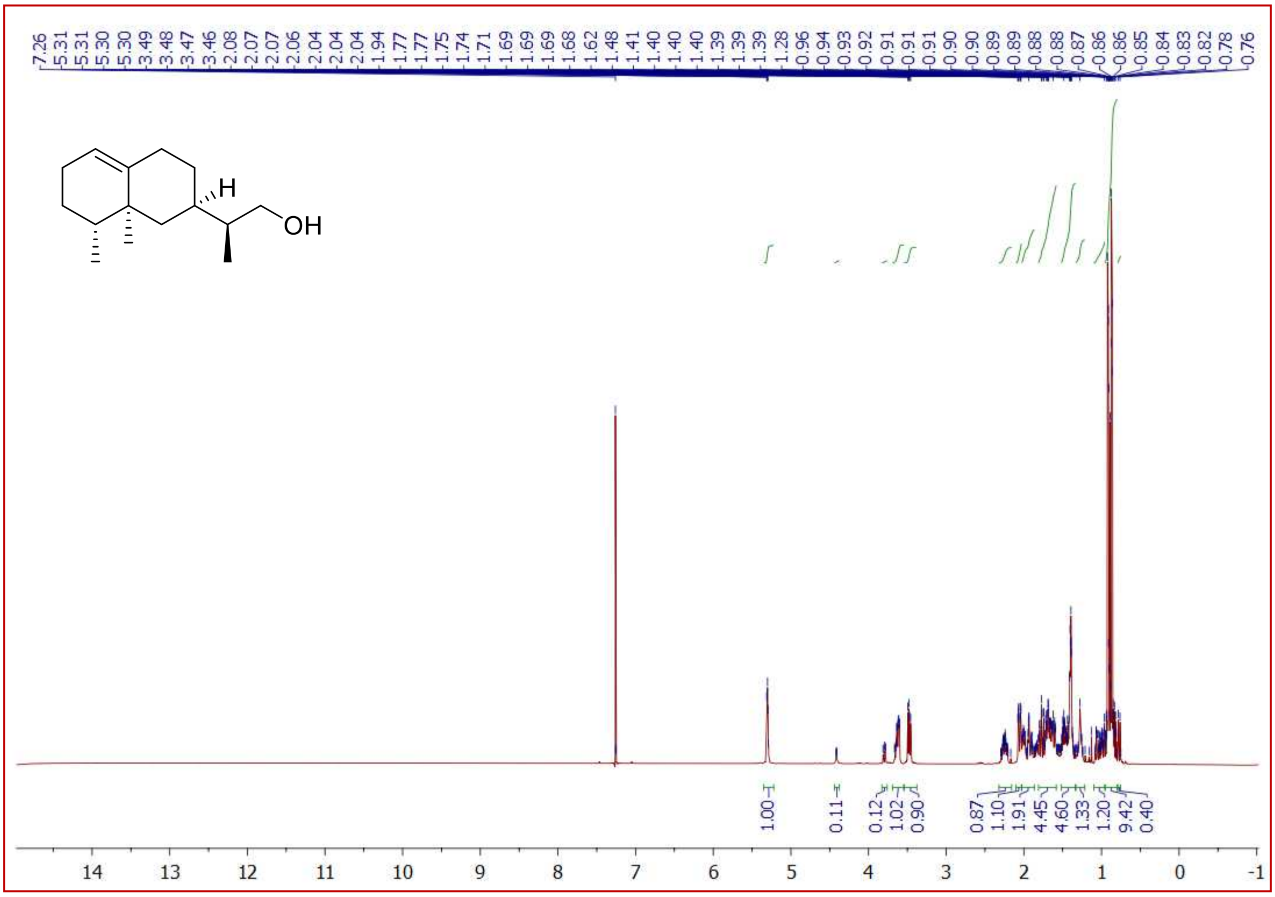

(*S*)-2-((2*R*,8*R*,8a*S*)-8,8a-Dimethyl-1,2,3,4,6,7,8,8a-octahydronaphthalen-2-yl)propan-1-ol (**8**), ^{13}C NMR

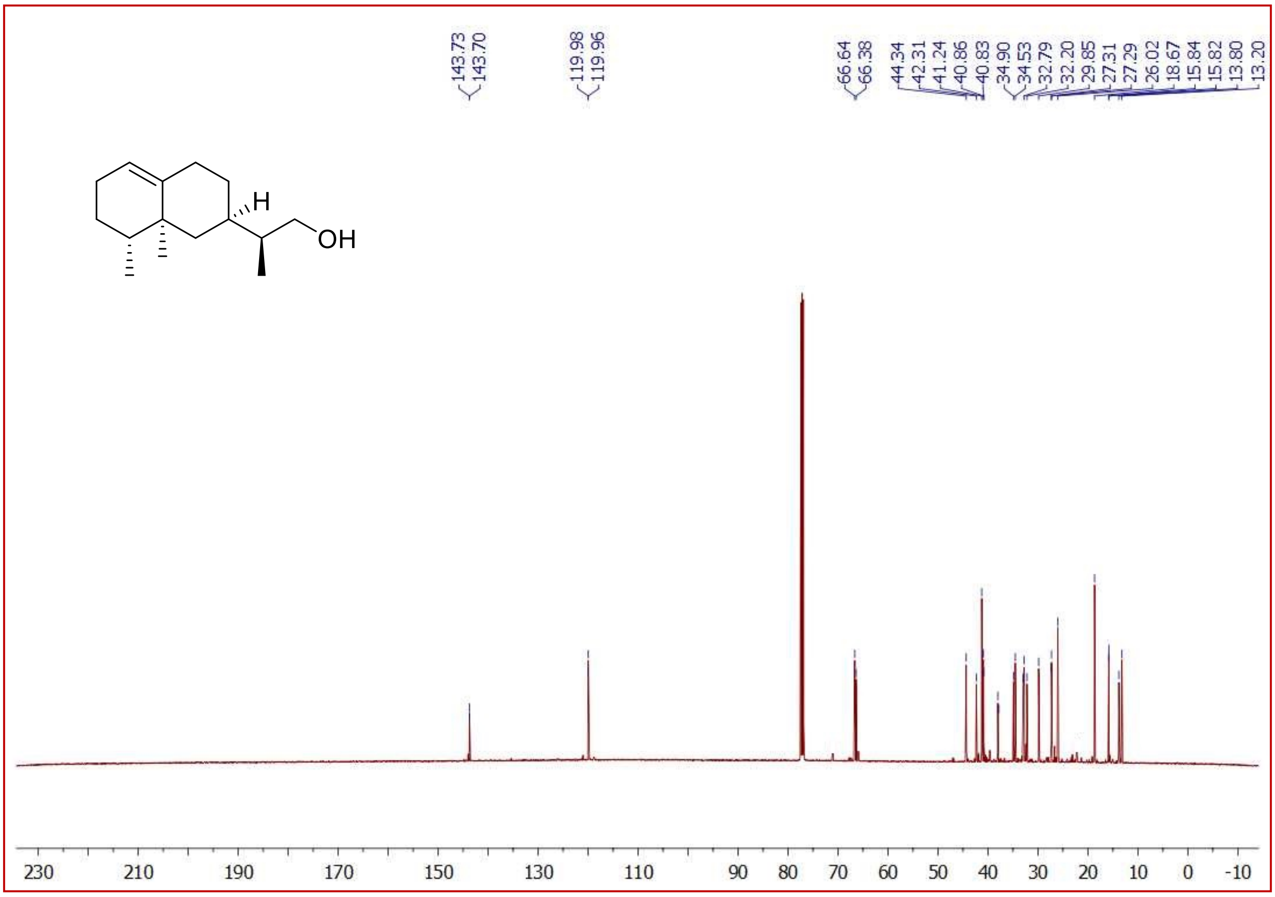

((1*S*,5*S*)-6,6-Dimethylbicyclo[3.1.1]heptan-2-yl)methanol (**9**), ^{1}H NMR

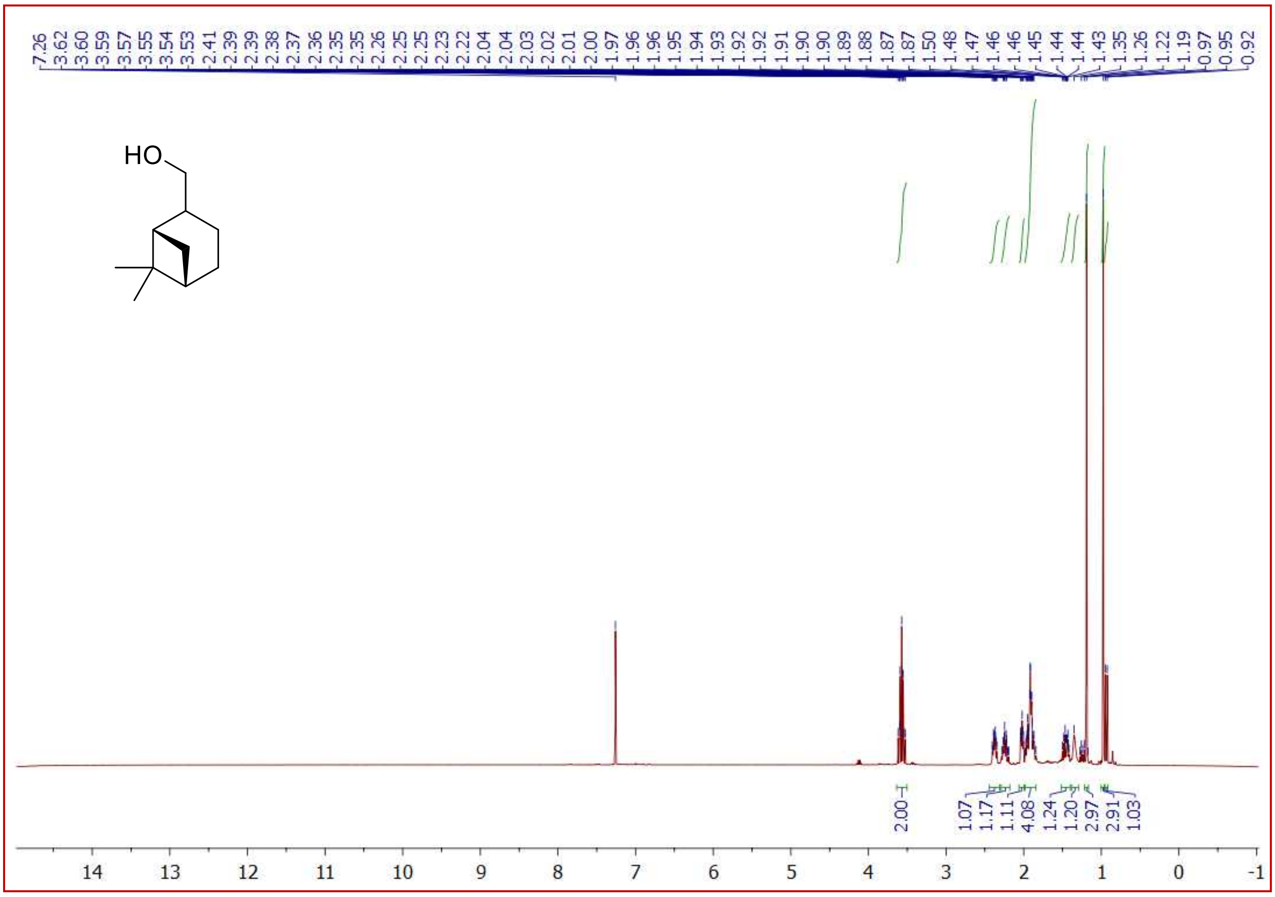

((1*S*,5*S*)-6,6-Dimethylbicyclo[3.1.1]heptan-2-yl)methanol (**9**), ^{13}C NMR

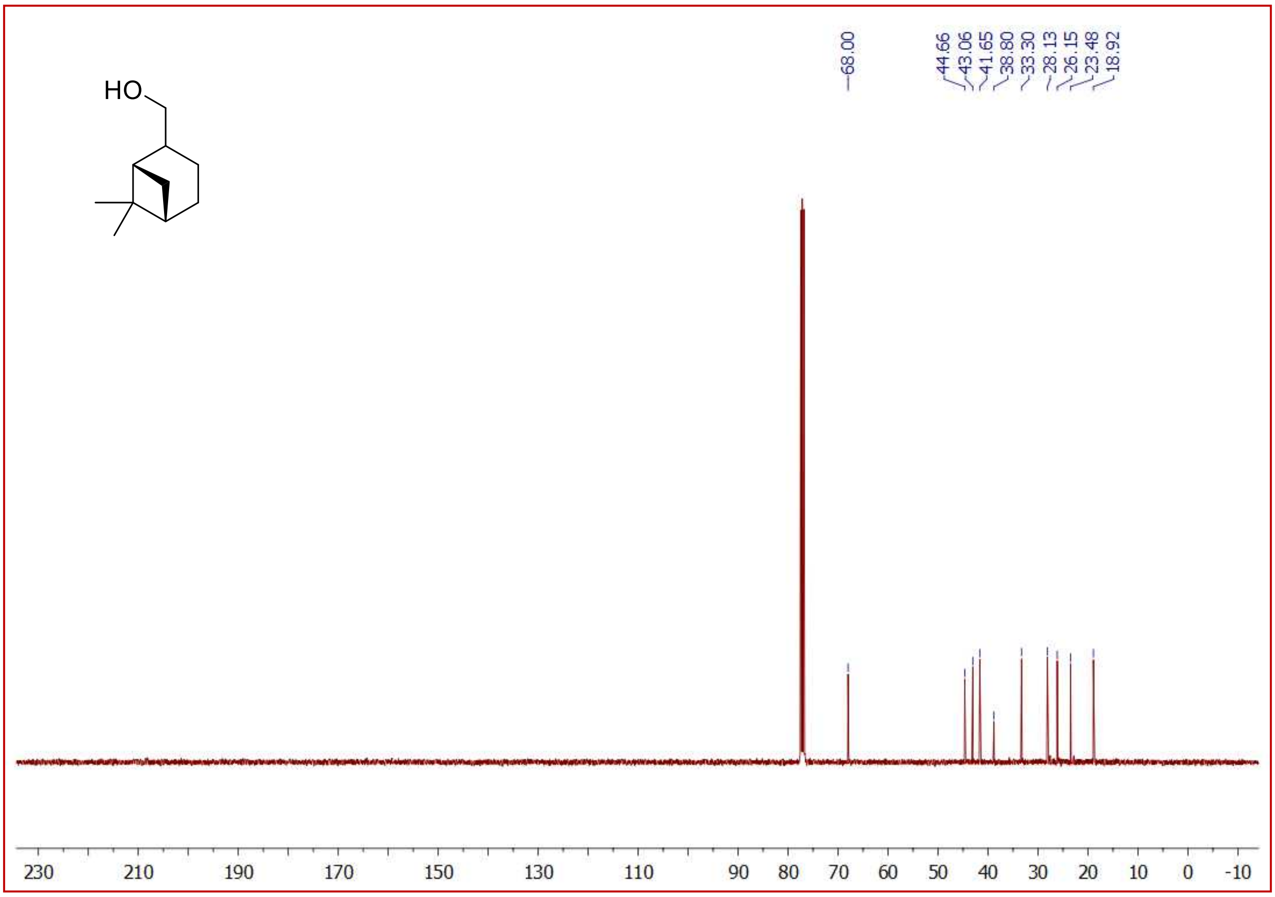

Decane-1,10-diol (**10**), ^{1}H NMR

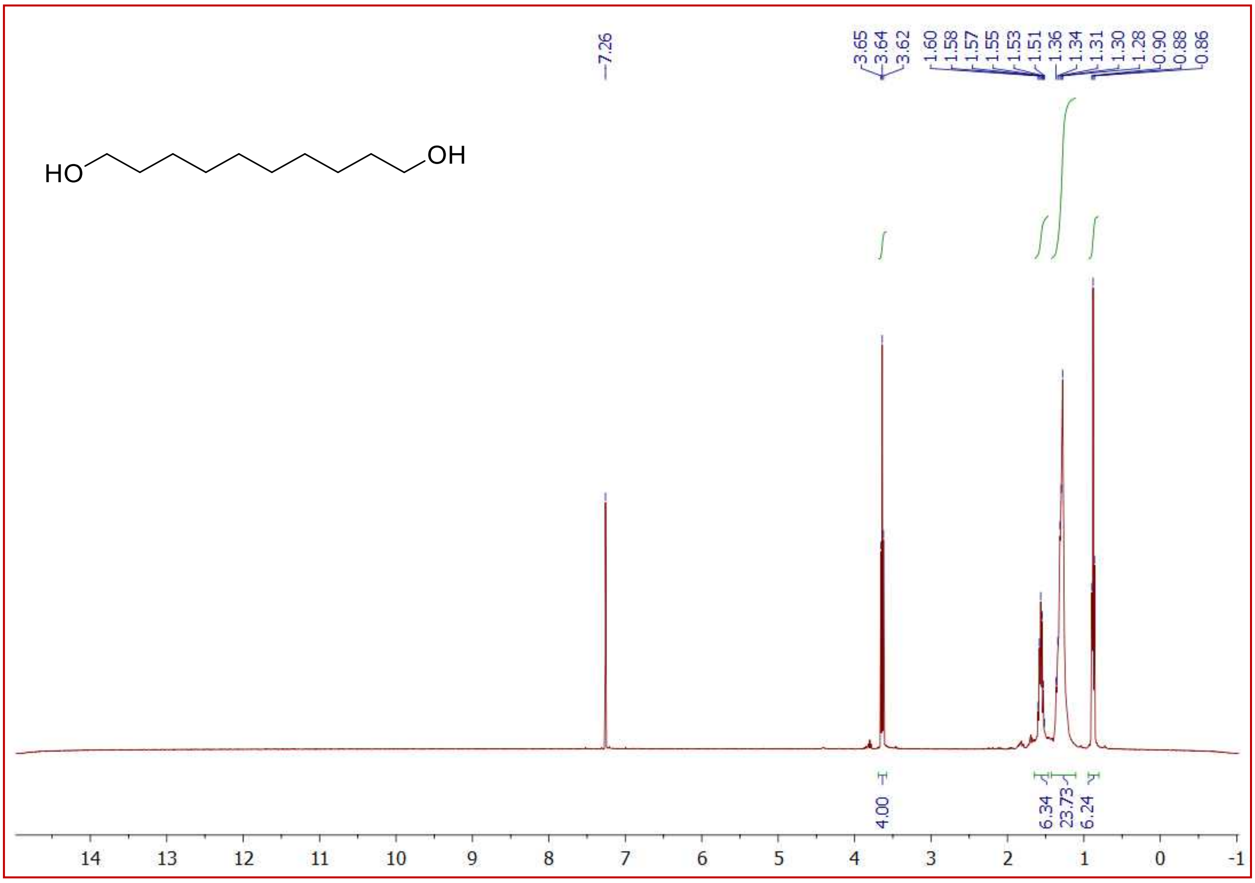

Decane-1,10-diol (**10**), ^{13}C NMR

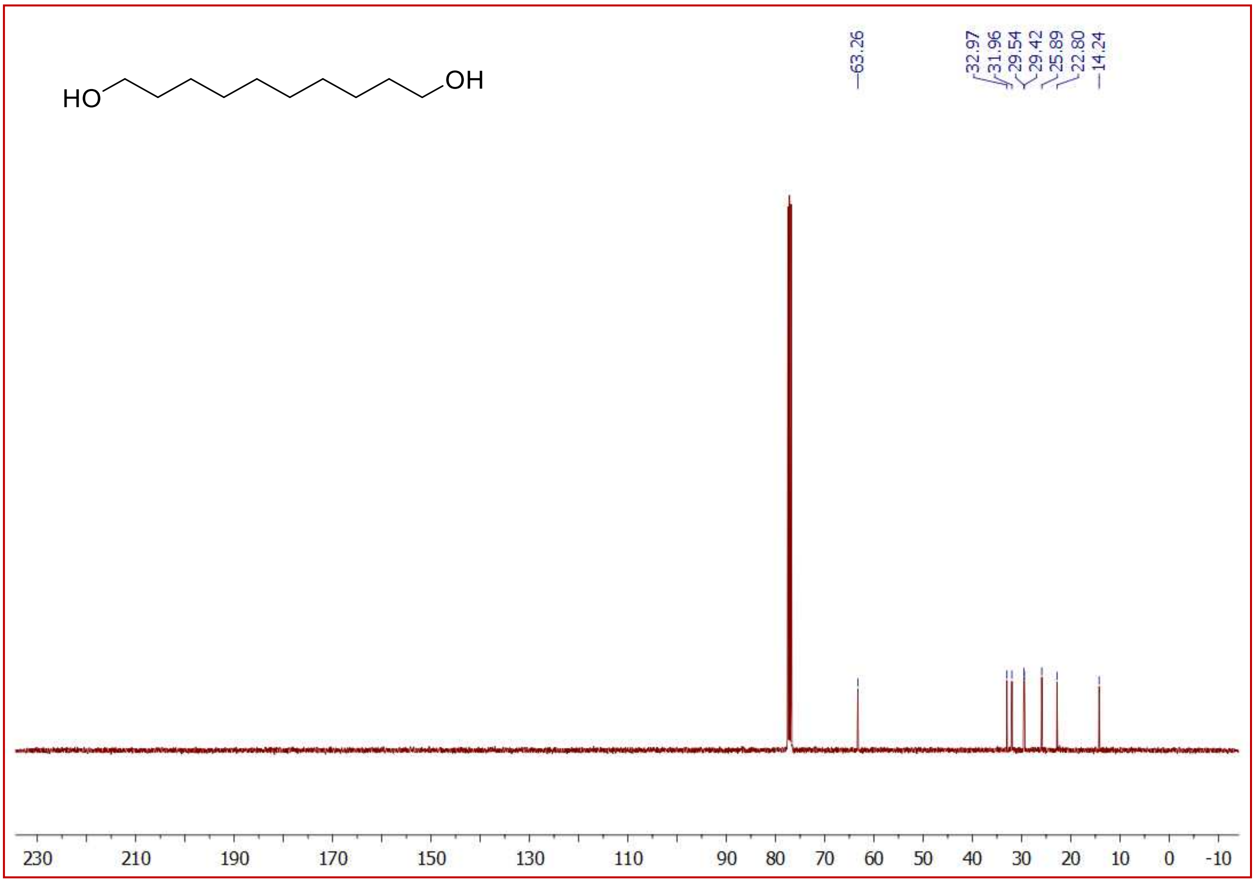

5-Bromopentan-1-ol (**11**), ^{1}H NMR

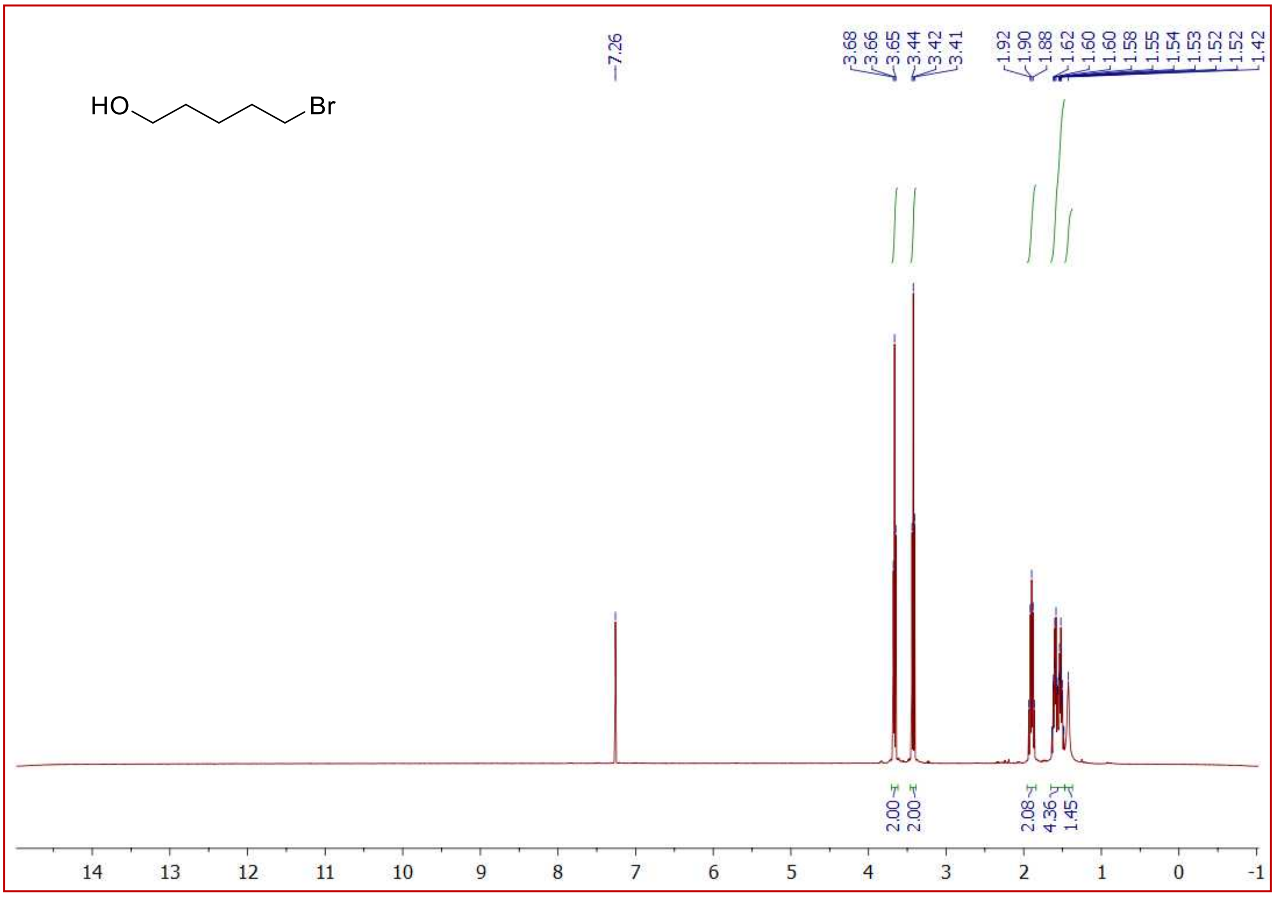

5-Bromopentan-1-ol (**11**), ^{13}C NMR

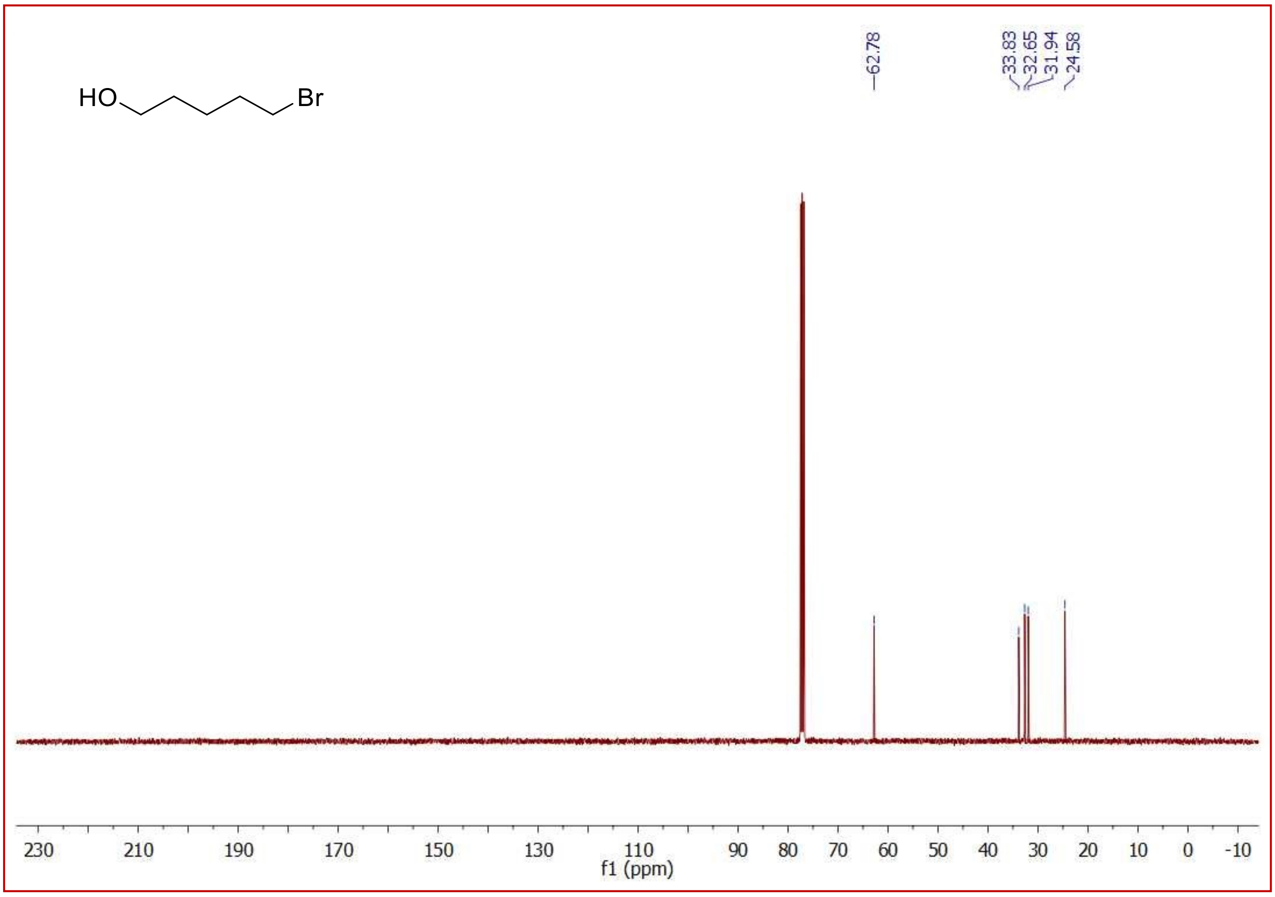

2-Phenylethan-1-ol (**12**), ^{1}H NMR

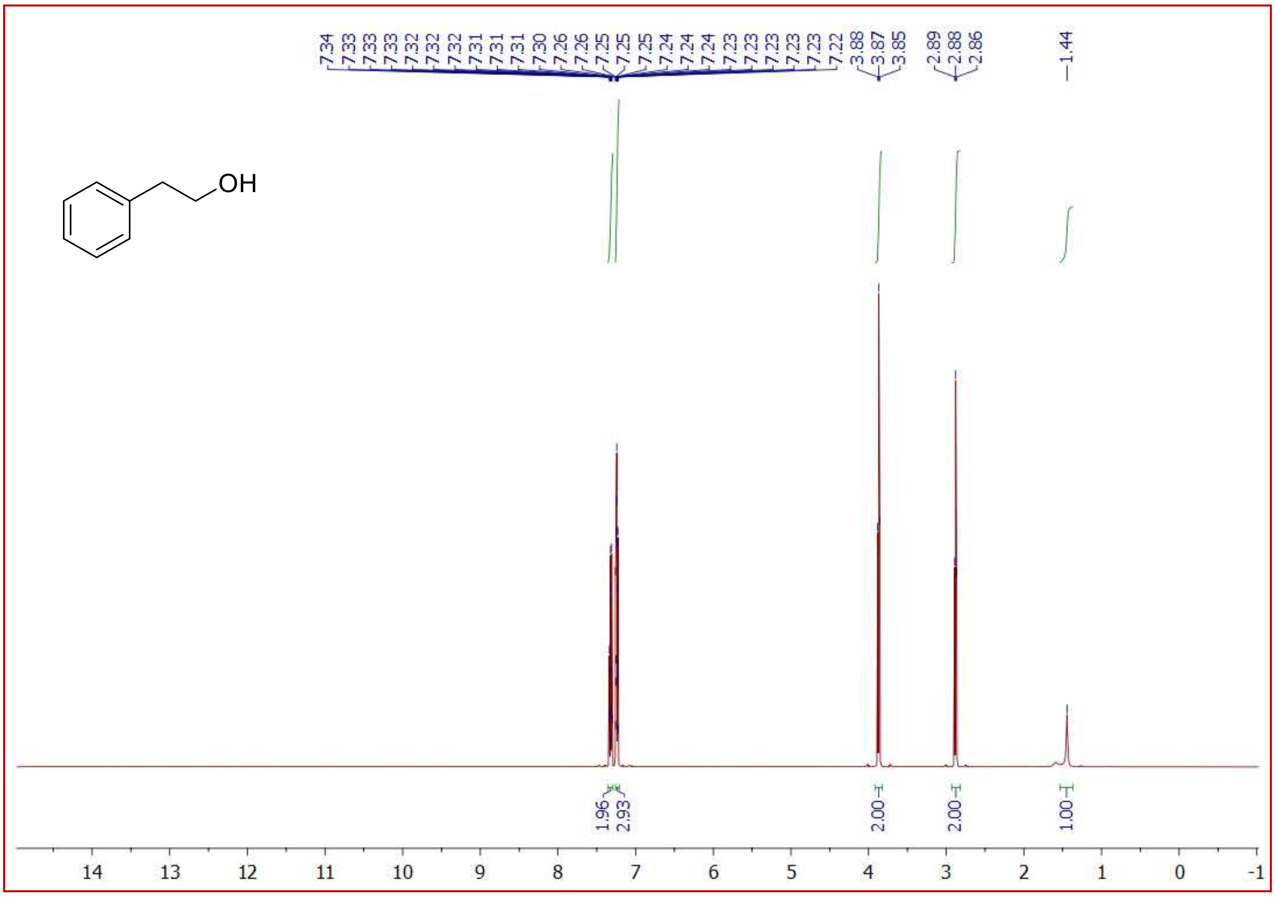

2-Phenylethan-1-ol (**12**), ^{13}C NMR

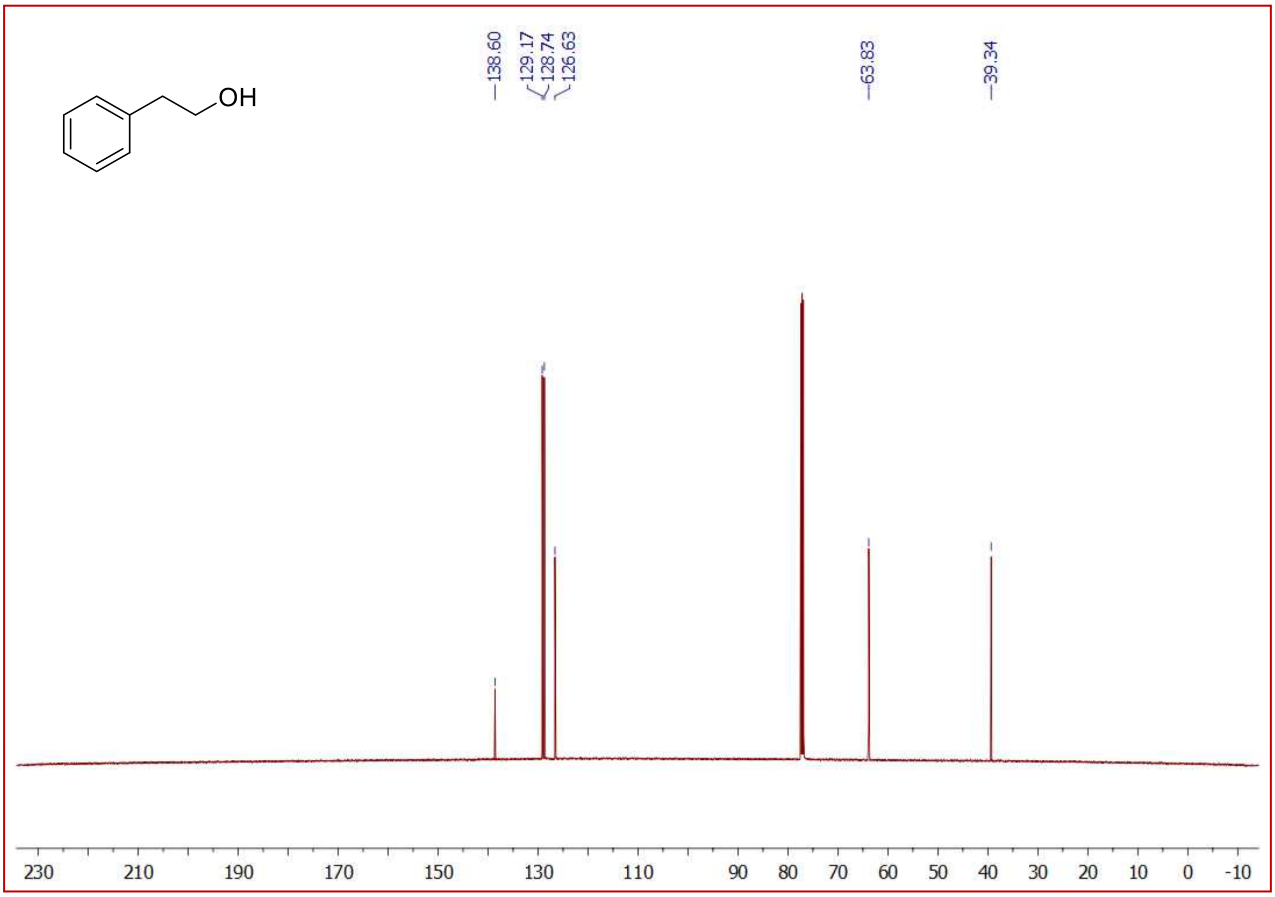

Supplement: Supplementary file 1 — Supplementary [file CHEM-26-11423-s001.pdf]
